# A Complete Protocol for Rapid and Low-Cost Fabrication of Polymer Microfluidic Chips Containing Three-Dimensional Microstructures Used in Point-of-Care Devices

**DOI:** 10.3390/mi10090624

**Published:** 2019-09-19

**Authors:** Trieu Nguyen, Aaydha Chidambara Vinayaka, Dang Duong Bang, Anders Wolff

**Affiliations:** 1 Department of Biotechnology and Biomedicine, Technical University of Denmark, DK 2800 Kgs. Lyngby, Denmark; 2 Laboratory of Applied Micro and Nanotechnology (LAMINATE), Division of Microbiology and Production, National Food Institute, Technical University of Denmark, Kemitorvet, Building 204, DK 2800 Lyngby, Denmark; viaa@food.dtu.dk (A.C.V.); ddba@food.dtu.dk (D.D.B.)

**Keywords:** polymer, microfluidic chips, point of care, three-dimensional microstructures, protocol

## Abstract

This protocol provides insights into the rapid, low-cost, and largescale fabrication of polymer microfluidic chips containing three-dimensional microstructures used in point-of-care devices for applications such as detection of pathogens via molecular diagnostic methods. The details of the fabrication methods are described in this paper. This study offers suggestions for researchers and experimentalists, both at university laboratories and in industrial companies, to prevent doom fabrication issues. For a demonstration of bio-application in point-of-care testing, the 3D microarrays fabricated are then employed in multiplexed detection of *Salmonella* (*Salmonella* Typhimurium and *Salmonella* Enteritidis), based on a molecular detection technique called solid-phase polymerase chain reaction (SP-PCR).

## 1. Introduction 

Lab-on-a-chip technology for biochemical applications such as bio-sensing, pathogen detection, and safety control has grown tremendously in recent years [1,2,3,4,5,6,7,8]. Most of the applications are on fluorescent bases, and thus require optical materials to fabricate the chip part. Materials for optical measurements need to meet strict criteria, namely low surface roughness and high transparency with visible light and UV light. In this scenario, microfabrication technique is mostly applied to glass and fused silica (silicon is not in this catalogue, since it is not transparent with visible light and UV light). Nevertheless, the materials mentioned above are expensive, and difficult to handle (not only on an industrial scale, but also on a small scale, such as for university laboratories). Over the last 20 years, due to the rapid development of material science, polymers that meet optical demands have been made available; for instance, Cyclo-olefin Polymer (COP) and Cyclic Olefin Copolymer (COC), Polystyrenes (PS), Poly(methyl methacrylate) (PMMA), etc., Micro-, nano-fluidic devices with 1D channels have been fabricated using these materials, for bio-physical measurement and sensing [9,10]. Converting these thermoplastic polymers into three-dimensional (3D) microstructures with low surface roughness and high production efficiency (high reproducibility) is, however, a challenging task, especially for large-scale and low-cost applications. We previously attempted to produce 3D structures with COC and PS for biosensing and pathogen detection [11,12]. However, there are no systematic reports or protocols that fully describe the fabrication steps and production efficiency, and that which could aid massive production on an industrial scale. In this report, we present a complete and systematic protocol of a largescale and highly reproducible technique, employed for rapid fabrication of 3D micro-array optical structures for rapid pathogen detection devices. This protocol, at large, can be extended and applied to the fabrication of any 3D microstructure (on-chip and off-chip), down to the order of 10 microns. Because the material used is thermoplastic, the industrial-scale production technique chosen is polymer injection moulding. The fabrication process includes three main steps: (i) designing of the insert (also called the shim) and simulation of the milling tools; (ii) shim fabrication by micro-milling; and (iii) polymer injection moulding. Ways to avoid mistakes in the fabrication processes are also explained. As a proof of bio-application, after the fabrication step, the resulting 3D micro-array (called supercritical angle fluorescence (SAF) structures) is used in a multiplexed SP-PCR to detect and differentiate *Salmonella* Typhimurium and *Salmonella* Enteritidis. 

## 2. Fabrication Protocol

### 2.1. Design the Shim and Simulate the Milling Tools

#### 2.1.1. Software

In order to construct the shim (the insert in the injection mould), we used Autodesk Inventor 2018, which is a computer-aided design (CAD) application for 3D mechanical design. For milling simulation and Computer-Aided Manufacturing (CAM), we use Cimatron E13. Both software are licensed to Technical University of Denmark (Lyngby, Denmark).

#### 2.1.2. Design the Shim

**Figure 1:**
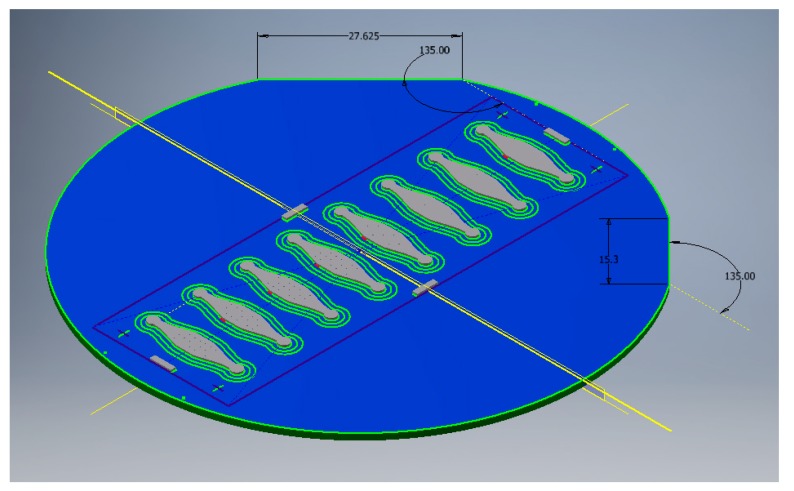
The CAD design of the shim (diameter = 85 mm). The number in the figure is in mm.

**Figure 2:**
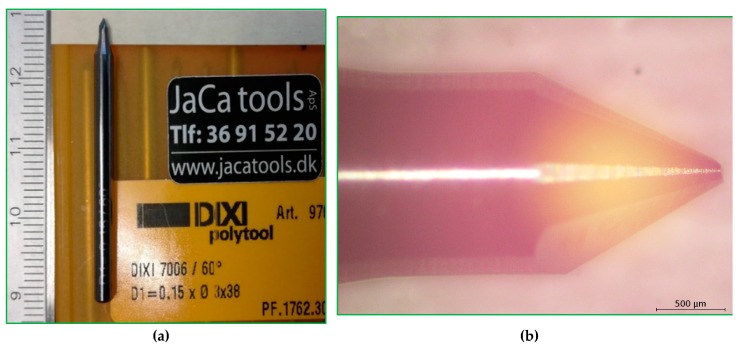
(**a**) Digital picture of the milling tool, DIXI 7006. (**b**) Microscopic picture of the tip of the milling tool (engraving tool ¾).

The injection-moulded insert (shim) had a diameter of 85 mm. For the case study, we fabricated the 3D microstructures that had a dimension of 100 microns. As aforementioned, the protocol itself can be applied for the fabrication of other structures, down to the order of 10 microns. The design and dimensions of the shim are shown in Figure 1. The top diameter of the microstructure was 100 micron, which is suitable for the milling tool, DIXI 7006 (Figure 2a). The microscope image of the milling tool is shown in Figure 2b.

#### 2.1.3. Simulation the Milling Tool

a. Bulk Milling

Bulk milling is used to rapidly remove most aluminum parts that are not critically small and accurate (typically for milling structures that are bigger than 500 µm). The surface formed after this step is rough and needs to be polished by using a smaller milling tip or a polishing paste. A 2-mm endmill tool is used for bulk milling.

b. Inclined Angle Milling

Inclined angle milling creates a slip angle, which aids in reducing friction during the demolding of the injection moulding process. DIXI 7009 with 30-degree angle is used.

c. Microarray Milling

The shim has an inverse structure to that of the micro-array. The injection-moulding step converts the inverse structures from the shim to the 3D microarray structure on the polymer chips. In order to create the inverse structure on the shim, we use DIXI 7006, a milling tool.

### 2.2. Micro-Milling the Shim

In order to gain low surface roughness and high dimensional accuracy of the structures on the resulting polymer chip, before performing the micro-milling, the aluminum plate needs to undergo surface polishing on one side, which is then chosen to be the back of the shim for injection moulding.

#### 2.2.1. Preparation 

**Figure 3:**
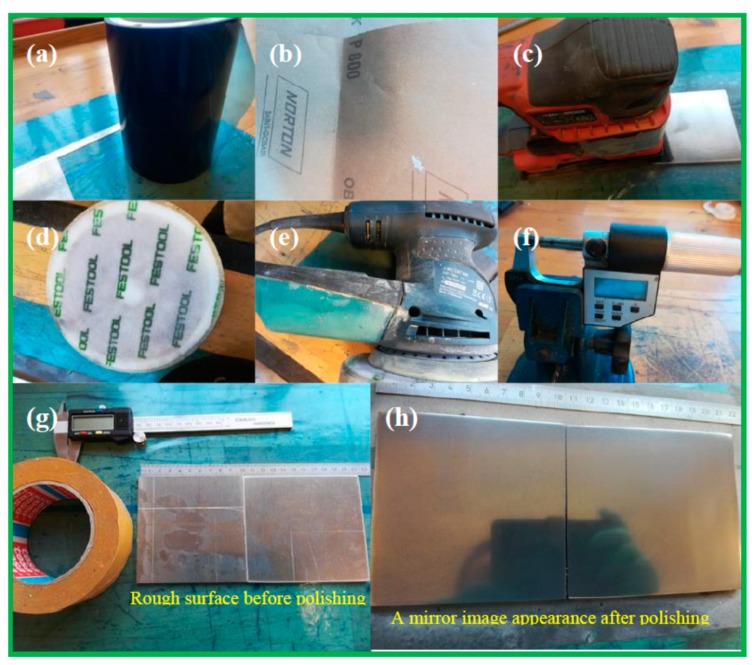
Digital pictures of the steps taken to polish the back of the Al plate. (**a**) Blue tape to cover the table surface and workplace; (**b**) sand paper Norton P 800; (**c**) Black Decker Duosand to sand the surface; (**d**) polishing sponge; (**e**) the Bosch GEX 125-1 AE machine that runs the polishing sponge; (**f**) Electronic IP54 Outside Micrometer; (**g**) and (**h**) show the Al plate, before and after polishing, respectively.

We used hard aluminum 2017 to make the shim. The aluminum sheets were cut into squares with a dimension of 100 × 100 mm^2^. The following steps were taken to polish the bottom of the aluminum sheet:

- The Al-plates were immobilized on a flat, smooth surface by applying a layer of double-sided tape (Figure 3a,g).
- Black Decker Duosand (Black Decker, Baltimore, MD, USA) with 800 grit wet and dry sandpaper (P800) was used for 15 minutes at maximal speed (Figure 3b,c).
- Autosol ALUMINUM polishing paste was applied to the aluminum sheet surface and a polishing sponge was attached to the Bosch GEX 125-1 AE machine (Bosch, Gerlingen, Germany), which was then run for 15 min at maximal speed (Figure 3d,e).
- The polishing paste residue was wiped off the aluminum surface with ethanol using cleanroom wipes, and then with cool water to observe a mirror finish (Figure 3h).
- The thickness of the aluminum sheet was measured using an Electronic IP54 Outside Micrometer (Figure 3f).

Figure 3h,g show the surfaces of the Al plates, before and after polishing.

#### 2.2.2. Milling Steps

- Place a sacrificial layer (PMMA 3 mm thick) at the bottom of the aluminum sheet to protect the milling stage and the milling tip.
- Determine the reference point, which is the centre (x = 0, y =0) of the aluminum sheet.
- Adjust the milling Z-stage position.
- Apply the milling oil.
- Start the milling.
- Change the milling tool.

#### 2.2.3. On-the-Fly Polishing Steps

After the bulk-milling step, we needed to polish the obtaining surface while the shim was still in the milling stage, hence called the on-the-fly polishing step.

**Figure 4:**
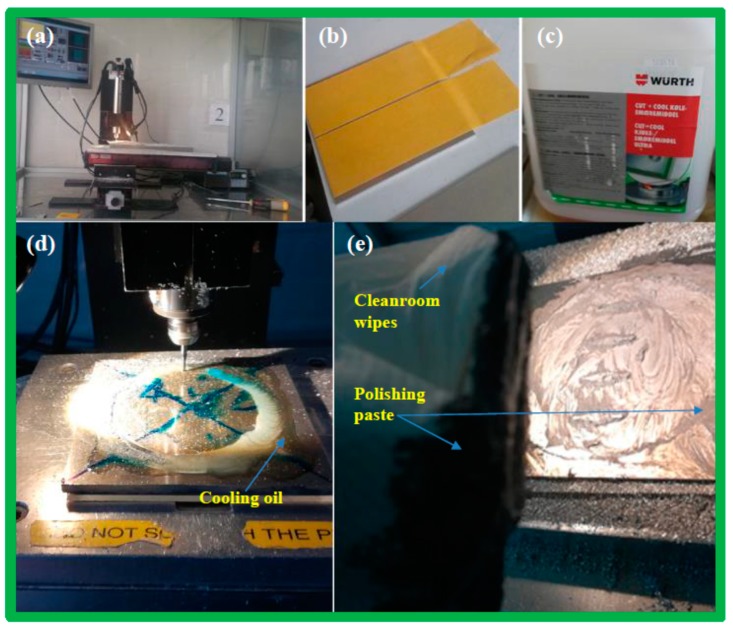
(**a**) Milling machine, Minitech Machinery Corporation, Norcross, GA, US (minimill 3); (**b**) the double-sided tapes that hold the Al plate in place during the milling stage; (**c**) cooling oil from Wurth, Künzelsau, Germany; (**d**) cooling oil is applied to the surface before the milling; (**e**) Autosol Aluminum polishing paste is applied after the bulk milling and cleanroom paper is used.

- Remove the aluminum debris from the surface using a vacuum cleaner, followed by water and a cleanroom paper, until the surface is completely clean. If the surface is not clean, the debris will cause scratches to the surface during polishing.
- Apply the Autosol polishing paste.
- Hand polish the surface of the reaction-chambers (which are formed after bulk-milling) by using a cleanroom wiper (Figure 4e).
- Redo the milling with the polishing paste still on the surface, to simultaneously polish the microarrays.

#### 2.2.4. Cleaning and Finishing

a. Clean the shim using cool water jet, followed by iso-propanol on the aluminum surface.

It is important to note the following: Do not try to remove the sacrificial PMMA layer (on the back of the shim) at this stage, since the adhesion between the PMMA layer and the aluminum sheet is strong due to the double-sided tape. Removing the PMMA layer will cause bending in the shim, and could result in a leaking problem doing polymer injection moulding.

b. Ultrasonic cleaning with acetone in 45 min; this will remove the remaining polishing paste from inside the micro-array’s holes and dissolve the adhesion of the double-sided tape. At this stage, it is safe to remove the PMMA sacrificial sheet by applying gentle force.

c. Another 30 min of ultrasonic cleaning with DI water to ensure that all the residues inside the micro-holes of the microarrays are removed.

**Figure 5:**
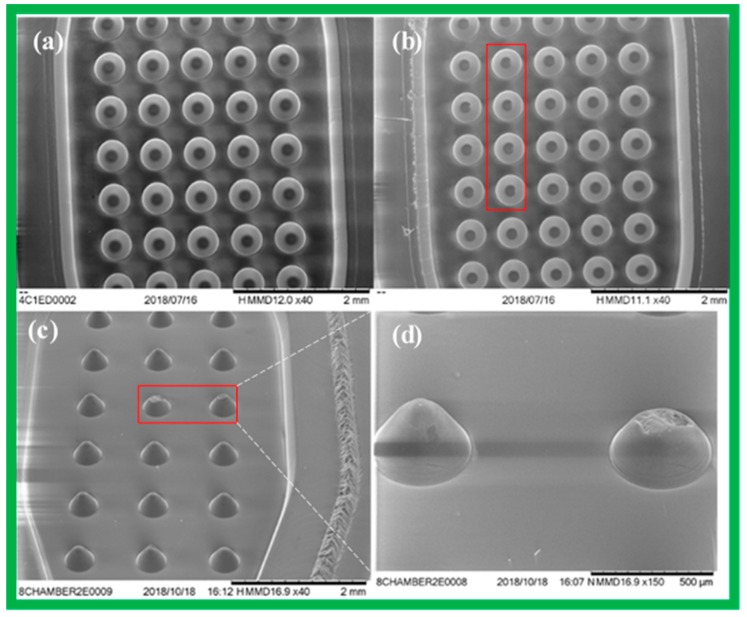
SEM images of: (**a**) Good 3D microstructures fabricated by polymer injection moulding using a milling shim, with enough washing time. (**b**) Defected structures caused by insufficient washing time (less than 30 min). (**c**) Defected structures when the shim did not have a washing step. (**d**) The enlargement of two defected microstructures shown in (**c**).

It is important to note that without the ultrasonic steps or complete removal of the residues, the micro-arrays will be damaged during polymer injection moulding (see Figure 5 for SEM images and a comparison). The ultrasonic cleaning step, therefore, helps eliminate failure and increases production efficiency of the fabrication and production.

### 2.3. Polymer Injection Moulding

#### 2.3.1. Alignment Marks

**Figure 6:**
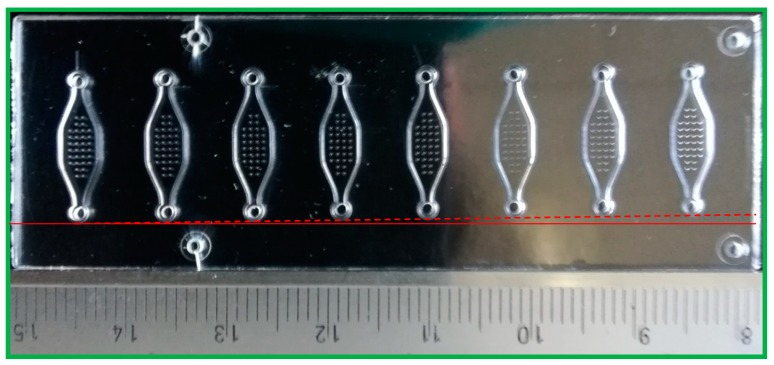
A polymer chip fabricated using a shim without alignment marks.

In order to align the shim in the injection mould, alignment marks are needed in the shim. Without alignment marks, the resulting chips can be asymmetric, and the misalignment in some cases can be seen by the naked eye (shown in Figure 6).

**Figure 7:**
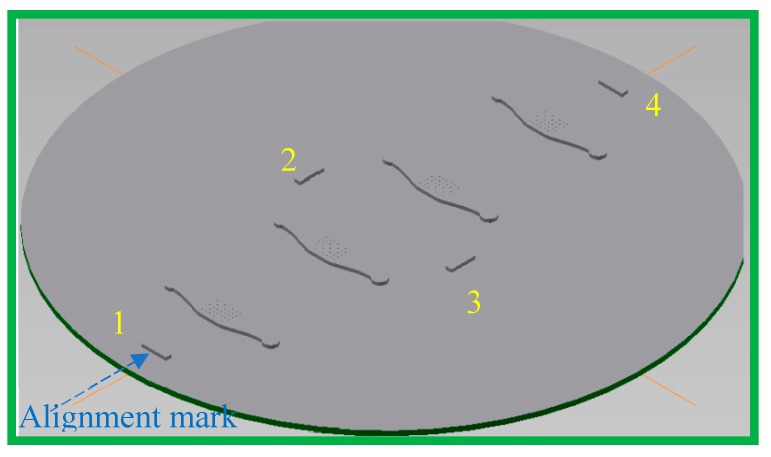
Injection moulding shim design with four alignment marks. The injection point is approximately at position 1 in the injection moulding machine.

Alignment marks, hence, increase fabrication efficiency and operation time. Figure 7 shows the design of a shim with four alignment marks.

a. Diesel Effect

**Figure 8:**
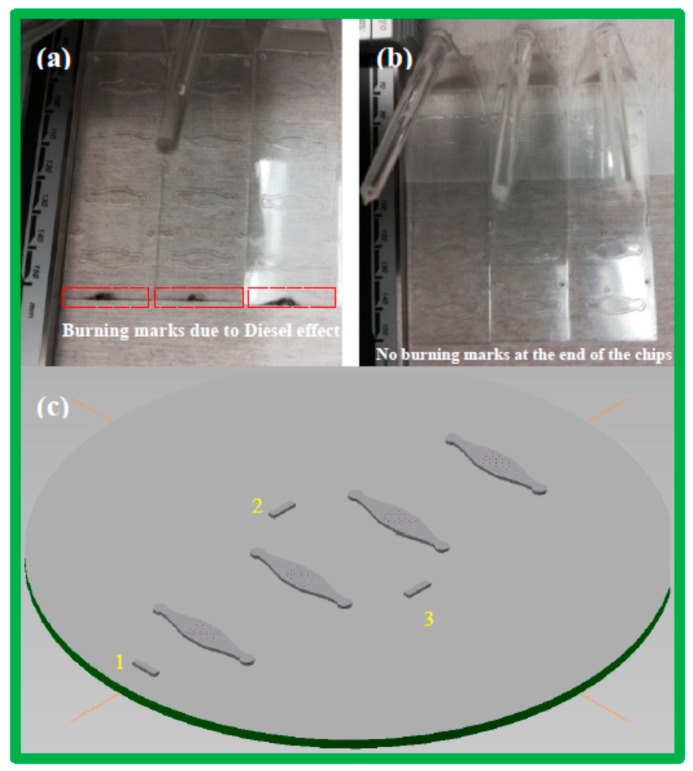
(**a**) Burn marks at the end of the chips due to the diesel effect. (**b**) Polymer chips without the diesel effect, as the shim used had only three alignment marks. (**c**) Shim with 3 alignment marks instead of 4 (as shown in Figure 7). The injection point is approximately at position 1 in the injection moulding machine.

Diesel effect occurs when air cannot escape during injection moulding, causing burn marks in the resulting sample. This effect especially occurs at the end of the flow path [13]. For instance, in our work (as shown in Figure 8a), the alignment mark was located at the end of the shim, causing trapped air and burn marks in the final chips.

b. Microscopic and SEM images when injection moulding to decide on mass production

After obtaining the first few polymer chips from a fabrication batch, it is important to inspect the chips under a microscope or an SEM to ensure that they qualify, i.e., they are well-aligned and well-shaped (see Figure 5 and Figure 6).

#### 2.3.2. Injection Moulding Parameters and Conditions

The protocol mentioned in the previous sections for rapid fabrication of an injection moulding shim can be used with a wide range of injection moulding machines. For this case study, injection moulding was carried out using a Victory 80/45 Tech hydraulic injection-moulding machine (Engel, Schwertberg, Austria) at the Technical University of Denmark. The chips shown in the previous sections were made from COC 5013L-10 (Topas Advanced Polymer GmbH, Frankfurt, Germany). The COC polymer used had a glass transition temperature (*T*g) of 135 °C. The injection temperature of the polymer was 270 °C, holding pressure time was 8 s, and the injection pressure was 800 bar.

In the injection moulding step, the important technical remark poses on the changeover step, which occurs when conducting injection moulding of two different kinds of polymers. When changing the materials (the type of polymer, such as from COC to PS), there must be a free-flowing (or cleaning) step, wherein the polymers are allowed to interchange from one to the other inside the injection moulding tool. This process can take approximately 20 to 30 min, depending on the amount of the previous residue.

**Figure 9:**
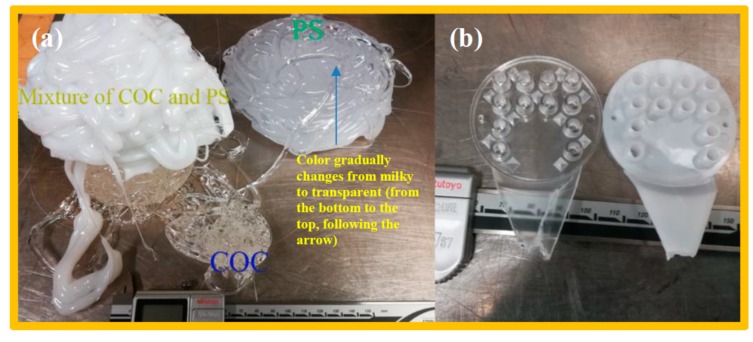
Color change of the injected polymers during the changeover from COC to PS. (**a**) The colour changes from transparent (COC) to milky (a mixture of COC and PS) to transparent again (PS). (**b**) If the injection moulding takes place during the transition stage, the resulting chip is milky in colour, as the chip on the right; the PS chip is on the left.

Figure 9 shows the colour changing step of the injected polymers doing the changeover from COC to PS. Figure 9a shows that the colour changes from transparent (COC) to milky (a mixture of COC and PS), and back to transparent again (PS). If the injection moulding takes place in the transition stage (without the cleaning step), the resulting chip is milky in colour; for instance, as shown in Figure 9b (the chip on the right).

This protocol is used to fabricate the shim insert for the production of 3D microstructures on polymer injection moulding chips. It was developed and applied at DTU-Nanotech (Technical University of Denmark, Kongens Lyngby, Denmark) in 2017–2018 (the department is now called DTU Health Technology). The protocol was firmly followed, and the efficiency (or reproducibility) of the producing injection moulding shim (insert) using micro-milling was observed to be 100%, assuming that the milling tip and milling machine are in good condition and function properly. Maintenance of the milling machines and use of new milling tips are recommended to guarantee the best results.

## 3. Application in Pathogen Detection

The protocol described in the previous sections for fabrication of an injection moulding shim containing 3D-microstructures with dimensions of 100 micron and high optical quality (low surface roughness due to two carefully polishing steps) can be extended to other 3D-microstructures that are used in optical and fluorescence measurements to detect pathogens or other targets in biological samples. As a case study, the 3D microstructure arrays fabricated in the previous section (SAF structures), such as that shown in Figure 5, can be employed in multiplexed detection of *Salmonella* (*Salmonella* Typhimurium and *Salmonella* Enteritidis), based on a molecular detection technique called Solid-Phase PCR (SP-PCR) [14]. The DNA probes used in this study to perform SP-PCR were previously reported by Hung et al., 2017 [14]. DNA probes (Table 1) diluted in 5× SSC (standard saline citrate) containing 0.004% Triton X were immobilized by precisely spotting on top of the SAF microlens array using sciFLEXARRAYER ultra-low volume dispensing systems (Scienion, Berlin, Germany). The microchip was exposed to UV irradiation at a wavelength of 254 nm for 10 min (Strataliner 2400, Stragtagene, CA, USA). Subsequently, the chip was dried using nitrogen and preserved under sterile dry conditions.

### 3.1. Detection of Salmonella Subtypes with SP-PCR

SP-PCR was performed in an SAF microlens array microchip to detect *Salmonella spp*. Before the SP-PCR, the reaction chamber was treated with 25 μL of 0.1× SSC buffer containing 0.25% bovine serum albumin (BSA) for 30 min at room temperature. SP-PCR was performed with 15 μL of PCR mixture containing 400 nM of *hil*A forward and 1600 nM of *hil*A reverse primers; 200 nM of *sdf* forward and 800 nM of *sdf* reverse primers; 600 nM of *fliC* forward and 2400 nM of *fliC* reverse primer; 2 ng of chromosomal DNA extracted from *Salmonella* Typhimurium *and Salmonella* Enteritidis; 400 ng BSA; 0.05 U/μL Phusion DNA polymerase; and 1× Phusion direct PCR buffer. The SP-PCR was conducted in a ProFlexTM 2× flat PCR system (Thermo Fisher Scientific). The PCR conditions were 94 °C for 5 min, following by 20 cycles of 94 °C for 10s, 60 °C for 20 s, 72 °C for 20 s, and another 15 cycles of 94 °C for 10 s, 65 °C for 20 s and 72 °C for 20 s. After the reaction, the chambers were washed with 1× SSC, followed by 1× SSC containing 1% Tween-20 for 10 s and 2 times with Milli-Q water for 20 s. The reaction chambers were dried at room temperature and the microchip was scanned using a BioAnalyzer 4F/4S scanner (LaVision BioTec GmbH, Bielefeld, Germany) with 200 ms exposure time.

### 3.2. Detection of Salmonella by SP-PCR on SAF Structures

**Figure 10:**
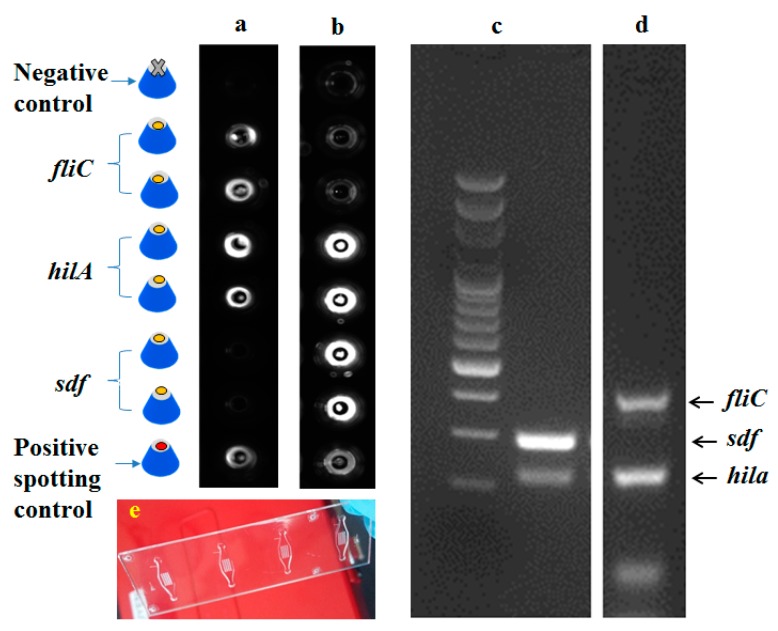
(**a**,**b**) Fluorescent images from the fluorescent scanner BioAnalyzer 4F/4S (LaVisionBiotec GmbH, Bielefeld, Germany) for multiplexed SP-PCR to detect and differentiate *S*. Typhimurium and *S*. Enteritidis. (**c**,**d**) The gel image of liquid phase PCR products. (**e**) Polymer microchip with an array of SAF structures.

SP-PCR amplification was performed directly on the SAF microlens array structures (Figure 10). The SP-PCR produced distinct fluorescence patterns on the SAF structures, confirming positive amplification of *hil*A gene-specific for *Salmonella spp*. Positive signals were also observed for both *fli*C and *sdf* genes, which confirmed serovar Typhimurium and serovar Enteritidis, respectively (Figure 10a,b). Further, identification of *Salmonella* at serovar level was made based on specific combinations of signals from these genes. The specificity and accuracy of the SP-PCR in the SAF-integrated microchip was confirmed with negative controls, wherein the fluorescence signals were very low compared to positive amplification signals from specific genes. Gel electrophoresis analysis of a left out reaction liquid from the SP-PCR revealed two specific amplicons of 238 bp of the *hil*A gene and 311 bp of the *sdf* gene for *S*. Enteritidis (Figure 10c). Similarly, specific amplicons of 238 bp of the *hil*A gene and 436 bp of the *fli*C gene for *S*. Typhimurium was shown (Figure 10d). There were no detectable amplicons in the negative control samples, inferring specificity of the primers for the target gene. In the SP-PCR, amplified products of the liquid phase reaction serve as templates. Thus, the fluorescence signals observed on the SAF structures after the SP-PCR were in accordance with the gel electrophoresis results.

### 3.3. Discussion on the Possible Point-of-Care Device Application

In view of a portable device, the transformation of lab-on-a-chip technology to point-of-care testing devices in term of instrumentation and electrical read-out can be addressed by utilisation of the open-source microcontroller platform, for instance, Arduino [15]. An open-source PCR machine was described by Josh Perfetto and Jessie Ho [16], where they set up a PCR thermocycler based on an Arduino Uno. The source code and a list of components are available for download, and it is also possible to buy an open-source PCR for 499 USD from their website. For the optical set-up of the device, one possibility is to use Lim’s design, as shown in [17].

## 4. Conclusions

In this paper, we reported a complete protocol for rapid, low-cost, and large-scale fabrication of 3D microstructures embedded inside a COC microfluidic chip. The resulting chips can be used for rapid detection of *Salmonella* using a conventional PCR machine or a point-of-care device. We provided details on fabrication steps and remarks to prevent doom fabrication mistakes. The fabrication method and protocol described in this paper can be extended to the production of any other 3D microstructure (both on-chip and off-chip) with dimension resolution in the order of 10 microns. This protocol provides insightful remarks, which can be of use to researchers and experimentalists, both at university laboratories and in industrial companies, especially for research and development (R&D).

## Figures and Tables

**Table 1:** List of primers used in this study [14].

Species	Target Gene	PCR Primers’ Sequences (5’–3’)
*Salmonella spp.*	*hil*A	*hil*A-F: GCG ACG CGG AAG TTA ACG AAG A
		*hil*A–R: Cy3-CAC GAT AGA GTA ATG CAG ACT CTC GGA TTG AAC CTG ATC
		*hil*A-solid phase surface probe: TTT TTT TTT TCC CCC CCC CCA AGA GCA TCG TTA
		CAT TGA AAC ACT GTA CGG ACA GGG CTA TCG GTT TAA TCG TCC GGT CG
*S. *Enteritidis	*sdf*	sdf-F: AAA TGT GTT TTA TCT GAT GCA AGA GG
		sdf-R: Cy3-TCT AAT GAA CTA CGT TCG TTC TTC TGG TAC TTA CGA TGA C
		sdf-solid phase surface probe: TTT TTT TTT TCC CCC CCC CCA TCA AAA AGG TTT AGT AAA TCA GCC TGT TGT CTG CTC ACC ATT CGC CAG CCA CCA CCT TC
*S. *Typhimurium	*fli*C	fliC-F: CCC CGC TTA CAG GTG GAC TAC
		fliC-R: Cy3-CTG CAG CGG GTT TTC GGT GGT TGT
		fliC-solid phase surface probe : TTT TTT TTT TCC CCC CCC CCA CTT ACG CTG CAA GTA AAG CCG AAG GTC ACA ACT TTA AAG CAC AGC CTG ATC TGG CGG AA
